# Poxvirus-encoded decapping enzymes promote selective translation of viral mRNAs

**DOI:** 10.1371/journal.ppat.1008926

**Published:** 2020-10-08

**Authors:** Fernando Cantu, Shuai Cao, Candy Hernandez, Pragyesh Dhungel, Joshua Spradlin, Zhilong Yang

**Affiliations:** Division of Biology, Kansas State University, Manhattan, Kansas, United States of America; University of California, Davis, School of Medicine, UNITED STATES

## Abstract

Cellular decapping enzymes negatively regulate gene expression by removing the methylguanosine cap at the 5’ end of eukaryotic mRNA, rendering mRNA susceptible to degradation and repressing mRNA translation. Vaccinia virus (VACV), the prototype poxvirus, encodes two decapping enzymes, D9 and D10, that induce the degradation of both cellular and viral mRNAs. Using a genome-wide survey of translation efficiency, we analyzed vaccinia virus mRNAs in cells infected with wild type VACV and mutant VACVs with inactivated decapping enzymes. We found that VACV decapping enzymes are required for selective translation of viral post-replicative mRNAs (transcribed after viral DNA replication) independent of PKR- and RNase L-mediated translation repression. Further molecular characterization demonstrated that VACV decapping enzymes are necessary for efficient translation of mRNA with a 5'-poly(A) leader, which are present in all viral post-replicative mRNAs. Inactivation of D10 alone in VACV significantly impairs poly(A)-leader-mediated translation. Remarkably, D10 stimulates mRNA translation in the absence of VACV infection with a preference for RNA containing a 5’-poly(A) leader. We further revealed that VACV decapping enzymes are needed for 5’-poly(A) leader-mediated cap-independent translation enhancement during infection. Our findings identified a mechanism by which VACV mRNAs are selectively translated through subverting viral decapping enzymes to stimulate 5’-poly(A) leader-mediated translation.

## Author summary

Decapping enzymes are encoded in eukaryotic cells and some viruses. Previous studies indicated that decapping enzymes are negative gene expression regulators by accelerating mRNA degradation and repressing translation. Surprisingly however, in this study we found that vaccinia virus (VACV) encoded-decapping enzymes, D9 and D10, are required to promote selective synthesis of viral proteins, although they are known to promote both cellular and viral mRNA degradation. We further showed that the unusual 5'-UTR of VACV mRNA, the 5'-poly(A) leader, confers an advantage to mRNA translation promoted by the decapping enzymes during vaccinia virus infection. Moreover, D9 and D10 are necessary for stimulating poly(A)-leader-mediated cap-independent translation enhancement during VACV infection. In the absence of VACV infection, D10 alone stimulates mRNA translation in a decapping activity-dependent manner, with a preference for mRNA that contains a poly(A) leader. The stimulation of mRNA translation by D10 is unique among decapping enzymes. Therefore, we identified a new mechanism to selectively synthesize VACV proteins through a coordination of viral mRNA 5’-UTR and virus-encoded decapping enzymes.

## Introduction

The 5′-methylguanosine (m^7^G) cap at the 5’ end of eukaryotic mRNA protects RNA from exonuclease digestion and facilitates recruiting translation machinery [1]. Decapping enzymes belong to the Nudix hydrolases superfamily of proteins containing a conserved Nudix motif that is critical for removing the mRNA 5’-cap by hydrolysis of the phosphodiester bond, resulting in a 5′-monophosphate RNA and a 7-methyl guanosine diphosphate [2]. The removal of 5’-cap leads to accelerated RNA degradation [3]. While Dcp2 was the first decapping enzyme discovered [4–7], more members of this family have been identified from various cellular and viral genomes [3, 8]. Decapping enzymes can reduce protein production by inducing mRNA degradation and interfering with mRNA translation processes. In eukaryotic cells, cap-dependent translation initiation and decapping are two competitive processes as they both need to access the 5’-cap. In addition to eliminating mRNA, the translation template, decapping enzymes also directly impede the translation process. Processing bodies (PBs) are distinct cytoplasmic foci of decapping machinery containing Dcp2, mRNAs, and other enzymes, in which mRNA translation is suppressed [9–13]. In yeast, the decapping can take place directly on polysomes [14, 15]. Dcp1, a decapping activator, physically interacts with the eIF4F translation initiation complex, which may interfere with translation initiation [16]. Two other decapping activators, Dhh1p and Pat1p, repress translation in a 5’-cap-independent manner. One likely mechanism is to slow the ribosome movement [17–19].

Poxviruses are a large family of viruses with dozens of known members. They cause many human and animal diseases, including zoonotic and deadly diseases. Viruses of this family have large, linear DNA genomes encoding hundreds of genes [20]. Vaccinia virus (VACV) is the prototypic member of poxviruses that was used as the vaccine to eradicate smallpox, one of the deadliest infectious diseases in human history [21]. VACV encodes two decapping enzymes, D9 and D10. D10 homologs are present in all members of the subfamily of chordopoxviruses, while D9 homologs are present in most members of chordopoxviruses. D9 and D10 induce both cellular and viral mRNA degradation [22–24]. The degradation of VACV-produced dsRNA by D9 and D10 also prevents the activation of host antiviral innate immune response [25, 26]. While the role of D9 and D10 in controlling mRNA turnover is well defined, their impacts on mRNA translation during VACV infection is largely unknown.

The 5’-poly(A) leader was initially discovered on several VACV post-replicative mRNAs over three decades ago [27–30]. We demonstrated that it is a universal feature in all VACV post-replicative mRNAs [31]. The poly(A) leader is generated by VACV RNA polymerase transcription slippage as it transcribes three constitutive T’s of the viral post-replicative gene promoter template strand (three constitutive As on the coding strand) [29, 32]. We and others independently discovered that the poly(A) leader gives to VACV post-replicative mRNAs a translational advantage in poxvirus-infected cells [33, 34]. How this unique cis-element of VACV post-replicative mRNAs promotes viral protein synthesis is yet to be determined.

Here in this study, we show that VACV decapping enzymes have an unexpected function to stimulate selective translation of viral mRNAs through the 5’-poly(A)-leader. Strikingly, D10 stimulates mRNA translation with a preference for mRNA with 5'-poly(A) leader. D9 and D10 together confer synergized advantage of the 5’-poly(A) leader-mediated translation during VACV infection. Therefore, although D9 and D10 decrease both viral and cellular mRNA levels, they promote selective synthesis of viral proteins by preferentially promoting viral mRNA translation.

## Results

### Inactivation of VACV decapping enzymes decreases overall translation activity in VACV-infected cells independent of PKR- and RNase L-mediated translational repression

**Fig 1:**
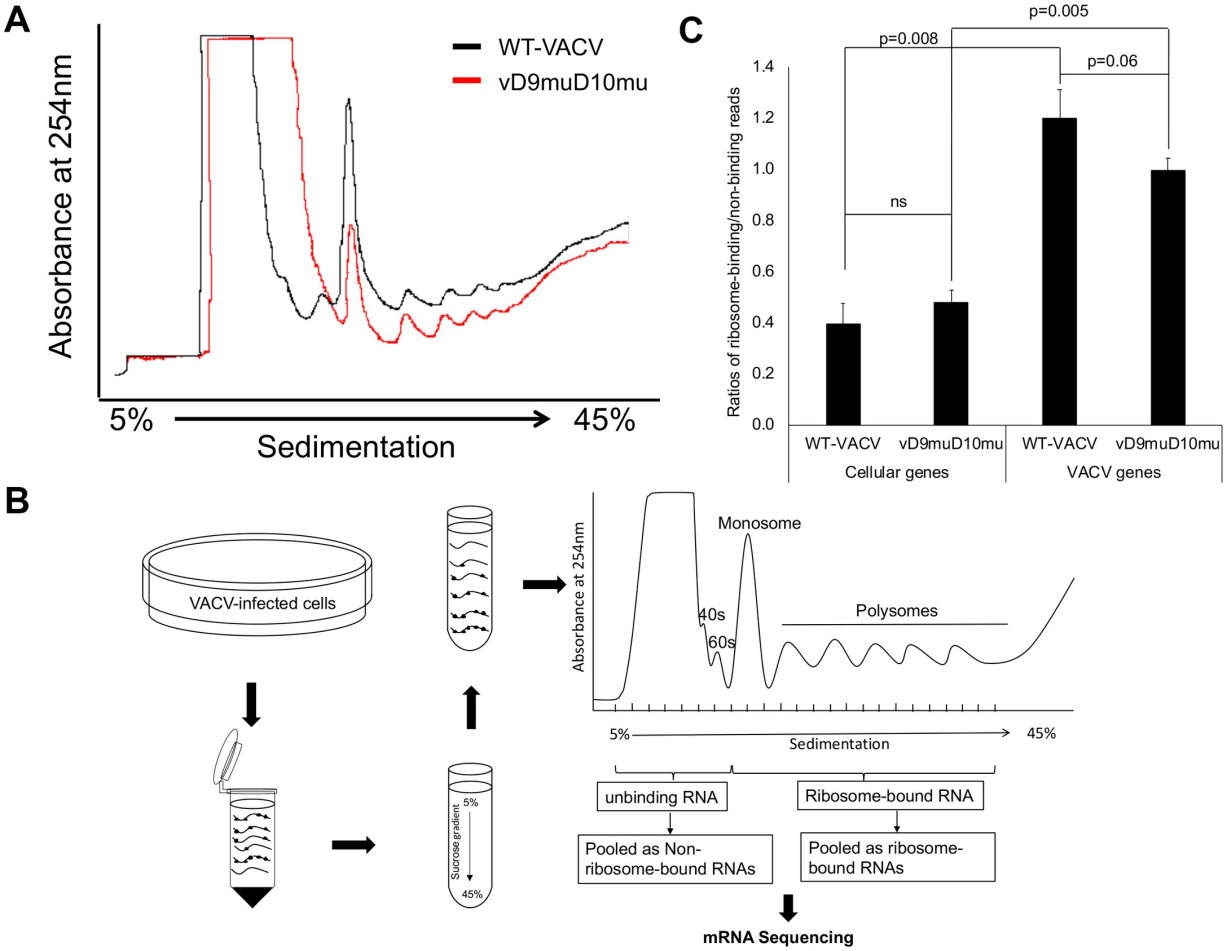
Inactivation of VACV decapping enzymes decreases overall translation activity in VACV-infected cells independent of PKR- and RNase L-mediated translational repression. **(A)** Polysome profiling to compare translation activities in WT- and vD9muD10mu-infected A549DKO cells. Cell lysates from virus-infected A549DKO cells at 8 hpi were applied to the sucrose gradient and fractionated using a fractionator. The decreasing trend in vD9muD10mu-infected A549DKO cells was reproducible with three biological replicates. **(B)** Experimental design of ribosome-binding/non-ribosome-binding RNA-Seq. **(C)** Global cellular mRNA translation activities in WT and vD9muD10mu-infected cells. The ratios of read counts of ribosome-binding RNAs to non-ribosome RNAs for cellular genes and viral genes in WT and D9muD10mu-infected cells were analyzed. Three biological repeats were carried out. P values are shown.

The Nudix motifs in both D9 and D10 were mutated to inactivate the decapping activity in the recombinant VACV vD9muD10mu [25]. Unlike WT-VACV, vD9muD10mu has a host range restriction partly because accumulating dsRNA activates PKR- and RNase L-mediated pathways, preventing replication in some cell types [26]. Nevertheless, it can replicate in A549 human lung carcinoma cells with PKR and RNase L knocked out (A549DKO). The A549DKO was generated using the CRISPR/Cas9 system. No residual PKR or RNase L could be detected [26], which we then confirmed (S1 Fig), and therefore the PKR and RNase L activation-related translation repression was excluded in A549DKO cells. However, vD9muD10mu still replicates at a lower level than wild type VACV in A549DKO cells [26], suggesting additional mechanism that hinders the mutant virus replication. To determine the impact of inactivating VACV decapping enzymes on mRNA translation during VACV infection, we compared global mRNA translation activities in vD9muD10mu- and wild-type (WT)-VACV infected A549DKO cells using polysome profiling. Eight hours post-infection (hpi) is a time point with active viral mRNA translation. Overlay of the polysome profiles from vD9muD10mu- and WT-VACV-infected A549DKO indicated a decrease in translation activity in vD9muD10mu-infected cells, evidenced by decreased polysomes and monosome that contain actively translating mRNAs (Fig 1A). Because the lack of decapping activity in vD9muD10mu-infected cells increases mRNA abundance in virally infected cells [25], the decrease in translational activity is not due to a decline of total mRNA level. This finding suggests a PKR- and RNase L-independent translational repression that results from VACV decapping enzyme inactivation during infection.

### VACV post-replicative mRNA translation is more severely suppressed in vD9muD10mu-infected cells

To investigate whether translational suppression of viral and cellular mRNAs is differentially affected in vD9muD10mu-infected cells, we sequenced mRNAs from ribosome-binding and non-ribosome-binding fractions in vD9muD10mu- and WT-VACV-infected A549DKO cells at 8 hpi, respectively, following the procedure depicted in Fig 1B. We included monosome-binding mRNAs in the ribosome-binding fraction as mounting evidence suggests that monosomes also actively translate mRNAs, especially mRNAs with short CDSs [35, 36], a feature of VACV ORFs (S2 Fig). We carried out three independent biological replicates, which were highly reproducible with correlation coefficiency square (R^2^) over 0.97 between any two replicates (S3 Fig). We used the ratios of ribosome-binding to non-ribosome-binding mRNAs as the indicator of relative translation efficiency. We did not observe an overall lower cellular mRNA translation efficiency in vD9muD10mu-infected cells than in WT-VACV infected cells (Fig 1C). The relative translation efficiency is lower in vD9muD10mu-infected cells for viral mRNAs, although the difference is not significant with a p-value of 0.06 (Fig 1C). Notably, we observed much higher relative translation efficiency for VACV mRNAs than cellular mRNAs (Fig 1C), consistent with our previous observation by ribosome profiling [33].

**Fig 2:**
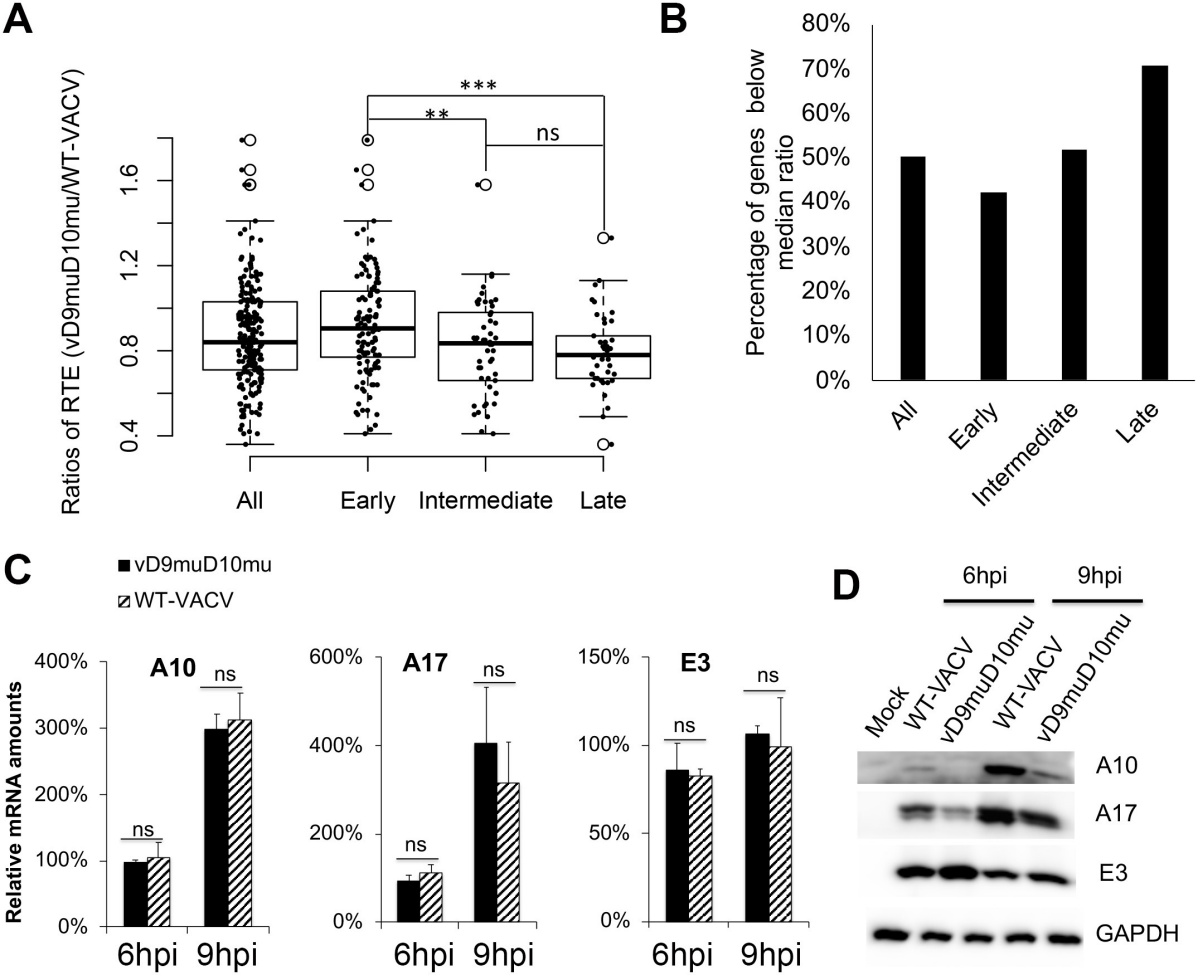
Translation of VACV early and late mRNAs is differentially affected by D9 and D10 inactivation during VACV infection. **(A)** Strip plot comparison of ribosome/non-ribosome-binding RNA ratios of early, intermediate, and late viral mRNAs in WT and vD9muD10mu-infected A549DKO cells. Solid dots represent the values of individual genes, which are overlaid on top of the corresponding whisker plot. ***<0.0001. **(B)** Percentages of genes below the median ratio of WT- to vD9muD10mu-infected A549DKO cells for early, intermediate, and late mRNA translation efficiency. **(CD)** mRNA (**C**) and protein levels (**D**) for E3, A10, A17 in WT-VACV- and vD9muD10mu-infected A549 DKO cells. The mRNA levels were determined by qRT-PCR and the protein levels were determined by Western Blotting analysis. ** indicates p ≤ 0.01, *** indicates p ≤ 0.001, ns indicates not significant.

VACV mRNAs are classified into three groups based on their temporal expression cascade: early (118 genes), intermediate (53 genes), and late (38 genes) classes [37–39]. The intermediate and late genes are collectively referred to as post-replicative genes as they are expressed after viral DNA replication. We analyzed the impacts of decapping enzyme inactivation on translation of the three classes of mRNAs by comparing the relative translation efficiency of VACV mRNAs in vD9muD10mu- and WT-VACV infected cells. As shown in Fig 2A, while there is an overall decrease of relative translation efficiency in vD9muD10mu-infected cells (median of ratio = 0.840 compared to WT-VACV), the extents of decrease were different among the early (median = 0.905), intermediate (median = 0.835), and late mRNAs (median = 0.780). The difference between the early and intermediate, as well as early and late, but not intermediate and late mRNAs, were statistically significant (Fig 2A). Further analysis indicated that 42% of the early mRNAs (49 out of 118), and 71% of late mRNAs (27 out of 38), had a lower than overall VACV mRNA median relative translation efficiency. The impact on the intermediate mRNAs was between early and late mRNAs (27 out of 53 had a lower than overall VACV mRNA median relative translation efficiency) (Fig 2B). These findings suggest a higher translation suppression effect on viral post-replicative mRNAs, especially late mRNAs, by viral decapping enzymes inactivation.

Further, the mRNA and protein levels of two late genes (A10 and A17) were compared in WT VACV- and vD9muD10mu-infected A549DKO cells. When they expressed similar levels of transcripts, the protein levels were much lower in vD9muD10mu infection (Fig 2C and 2D). In contrast, the protein levels of the E3 gene (an early gene) were similar in vD9muD10mu and WT-VACV infection, when the levels of mRNAs were equivalent (Fig 2C and 2D). These results confirmed that VACV late mRNAs are more dependent on the decapping activity of D9 and D10 for efficient translation, independent of PKR and RNase L activation-mediated translation repression.

### Inactivation of D9 and D10 decapping activities impairs selective translation of mRNA with a poly(A) leader during VACV infection

**Fig 3:**
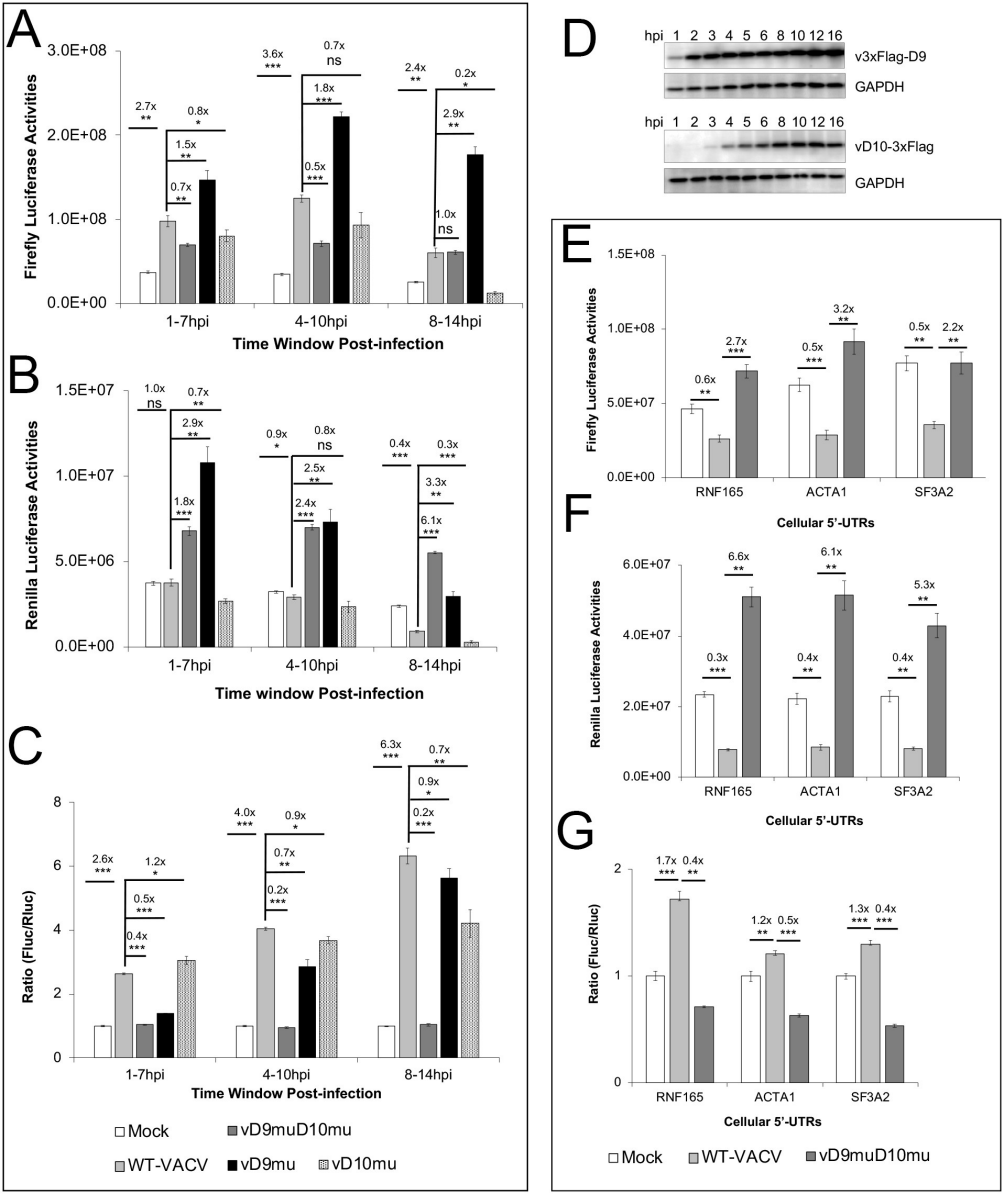
Inactivation of viral decapping enzymes substantially impairs selective translation of mRNA with a poly(A) leader during VACV infection. **(A-C)** Co-transfection of m^7^G-capped 12A-Fluc and Kozak-Rluc in WT, vD9muD10mu, vD9mu, and vD10mu-infected A549DKO cells at indicated time post-infection. Luciferase activities were measured 6 h post-transfection to evaluate the translation activities during the periods 1–7, 4–10, and 8–14 hpi, respectively. Fluc (**A**), Rluc (**B**), and Fluc/Rluc ratios with mock-infected samples normalized to 1 (**C**) are presented. **(D)** D9 and D10 expression over the course of VACV infection of A549 DKO cells at MOI 5. v3xFlag-D9 in which WT-D9 is tagged with a 3xFlag at N-terminus, and vD10-3xFlag in which WT-D10 is tagged with a C-terminal 3xFlag, were used, respectively. **(E-G)** Co-transfection of m^7^G-capped RNF165-, ACTA1- or SF3A-Fluc together with Kozak-Rluc in mock, WT-VACV-, and D9muD10mu-infected A549DKO cells at 8 hpi, respectively. Luciferase activities were measured 6 h post-transfection. Fluc (**E**), Rluc (**F**), and Fluc/Rluc ratios with mock-infected samples normalized to 1 (**G**) are shown. Error bars represent the standard deviation of 3 biological replicates. Significance determined by students *t-test* where p>0.05 (ns), p≤ 0.05 (*), p≤0.01 (**), and p≤0.001 (***). Numbers above significance represent fold change between compared samples.

While various cis-elements can regulate mRNA translation, a prominent difference between VACV early and intermediate/late mRNAs is that all the intermediate/late mRNAs have 5’-poly(A) leaders of heterogeneous lengths. The most frequent lengths are 11–12 A’s for late mRNAs and 8–9 A’s for intermediate mRNAs [31]. We previously showed that a 5’-poly(A) leader could confer a translational advantage in VACV-infected cells with 12 A’s having the maximal translational advantage [33]. Using a synthesized mRNA transfection-based reporter assay [40], we found that the decapping enzyme activities were required for selective translation of mRNA with a 5’-poly(A) leader (Fig 3A–3C). We co-transfected *in vitro* synthesized, m^7^G-capped, 12A-headed Firefly Luciferase (12A-Fluc), and Kozak sequence-headed Renilla Luciferase (Kozak-Rluc) mRNAs into mock, WT-VACV, and vD9muD10mu-infected A549DKO cells at 1, 4, and 8 hpi, respectively. Luciferase activities were measured 6 h post-transfection of reporter RNA to evaluate the translation activities during the periods 1–7, 4–10, and 8–14 hpi, respectively. The translation of 12A-Fluc RNA significantly decreased in vD9muD10mu-infected cells compared to WT-VACV-infected cells during 1–7 hpi and 4–10 hpi (Fig 3A). Translation of Kozak-Rluc RNA was not impaired, but increased, in vD9muD10mu-infected cells (Fig 3B). Interestingly, in WT-VACV-infected cells, Kozak-Rluc mRNA translation was not substantially affected before 10 hpi, but notably decreased during the 8–14 hpi time window (Fig 3B). The 12A-Fluc to Kozak-Rluc ratio is an indicator of the relative translational advantage of the poly(A)-headed RNA over Kozak sequence-containing 5'-UTR. Analysis of the ratios showed that the poly(A) leader conferred an increasing translational advantage with the progression of WT-VACV infection, while the advantage was abolished entirely in vD9muD10mu-infected cells (Fig 3C). The difference in luciferase activities were not due to the difference of transfected RNAs, evidenced by similar levels of Fluc in the mock, VACV, vD9muD10mu-infected cells (S4 Fig). Rluc RNA levels were also similar in the mock, VACV, vD9muD10mu-infected cells (S4 Fig). Similar analyses of three non-poly(A) leader 5’-UTR of cellular mRNAs, RNA165, ACTA1, and SF3A2, indicated that the inactivation of the decapping enzymes had a more substantial effect to reduce translation of mRNA with an poly(A) leader (Fig 3E–3G). Similar results to the cellular 5’-UTRs were observed using an mRNA with a precise deletion of the 12A’s used in Fig 3A–3C (S5 Fig). These findings indicated that VACV decapping enzymes are required for the 5’-poly(A) leader-mediated selective translation of VACV mRNAs during infection.

### Distinct effects by individual inactivation of D9 and D10 decapping activities on 5’-poly(A)-headed mRNA translation

We then examined the individual effects of D9 and D10 inactivation on poly(A)-leader-mediated selective translation in VACV infection using vD9mu and vD10mu, respectively. The inactivation of D9 rendered increased luciferase activities for both 12A-Fluc and Kozak-Rluc mRNAs (Fig 3A and 3B), either due to higher translation activities or decreased RNA degradation. In contrast, the inactivation of D10 rendered decreased luciferase activities for both 12A-Fluc and Kozak-Rluc mRNAs (Fig 3A and 3B), suggesting that D10 is required for efficient mRNA translation for both poly(A) leader and non-poly(A) leader-mediated mRNA translation (Fig 3A and 3B). While the ratios of 12A-Fluc to Kozak-Rluc in cells infected with either vD9mu or vD10mu also decreased to some extent at different times of infection, the highest decrease occurred in cells with vD9muD10mu infection, suggesting an optimal translational advantage of 12A-Fluc requires both decapping enzymes (Fig 3C). D9 is an early gene expressed immediately after VACV enters the cell, while D10 is an intermediate gene expressed after viral DNA replication [23, 37, 38, 41]. In A549DKO cells infected with VACV at an MOI of 5, D9 protein started to be detected at 1–2 hpi and increased over the course of infection, while D10 protein started to be detected at 3–4 hpi and reached its maximal expression at 12 hpi (Fig 3D). The effects of D9, D10, or their combined effect on translation were in line with their expression kinetics with higher effect late during infection (Fig 3D).

### D10 promotes mRNA translation in the absence of VACV infection

**Fig 4:**
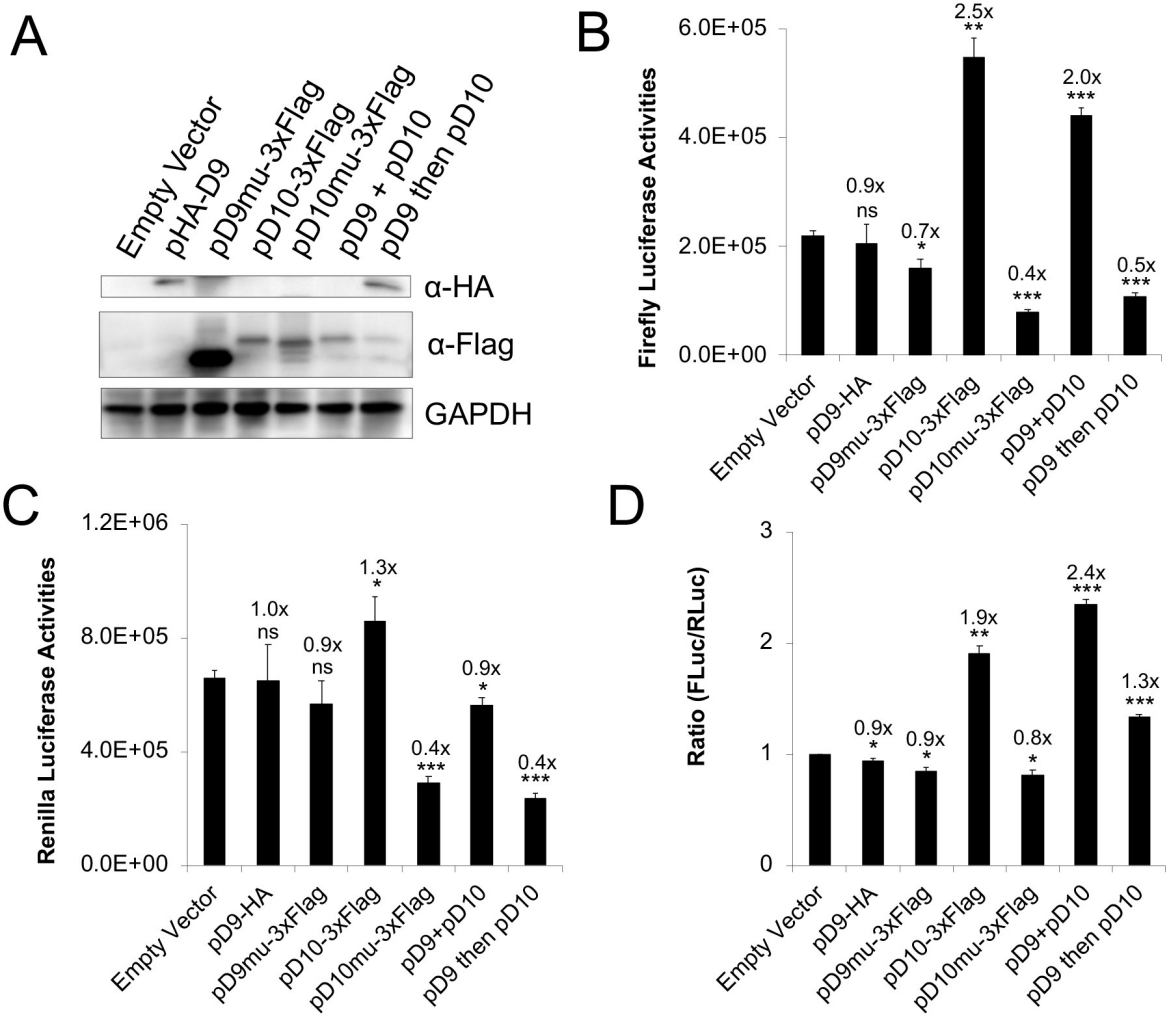
Expression of D10 in uninfected cells stimulates mRNA translation. **(A)** Western blotting analysis of D9, D10, and their decapping-inactive mutants in 293T cells transfected with corresponding plasmids using indicated antibodies. **(B-D)** 293T cells were transfected with indicated plasmids. 42 h post-transfection (in pD9 then D10, D10 plasmid was transfected 24 h after D9 plasmid was transfected), *in vitro* synthesized, m^7^G-capped 12A-Fluc, and Kozak-Rluc were co-transfected into the 293T cells. Luciferase activities were measured 6 h post RNA transfection. Fluc (**B**), Rluc (**C**), and Fluc/Rluc ratios with the empty vector normalized to 1 (**D**) are presented. Error bars represent the standard deviation of 3 replicates. Significance determined by students *t-test* where p>0.05 (ns), p≤0.05 (*), p≤0.01 (**), p≤0.001 (***). The numbers above significance represent fold changes. Significance and fold change were compared to the empty vector.

Our data indicated that inactivation of D10 suppressed both poly(A)-leader- and non-poly(A) leader-mediated translation (Fig 3), suggesting that D10 may stimulate translation. The finding is very intriguing, as many studies showed that decapping enzymes repress translation [9–19]. Next we examined the impacts of D9 and D10 expression on mRNA translation in uninfected cells. D9 and D10 coding sequences that were codon-optimized for expression in human cells were cloned into a plasmid with N-terminal HA (D9) and C-terminal 3xFlag (D10) tags, respectively, under the control of a CMV promoter. In multiple cell lines tested, the highest expression levels were observed in 293T cells (Fig 4A). Notably, in uninfected 293T cells, exogenous expression of D10 significantly enhanced translation of m^7^G-capped 12A-Fluc and Kozak-RLuc mRNA by 2.5- and 1.3-fold, respectively (Fig 4B and 4C). The ratio of 12A-Fluc to Kozak-Rluc indicated a significant 1.9-fold advantage for the poly(A)-leader-mediated translation in cells expressing D10 (Fig 4D). Interestingly, co-transfection of D9 and D10 did not dramatically increase the translational advantage (2.4-fold) (Fig 4B–4D), likely because D9 expression was reproducibly not detectable when co-transfected with D10 (Fig 4A). To mimic temporal expression nature of D9 (early) and D10 (intermediate) during VACV infection, we transfected D9 first then D10 to avoid the dominant repression effect of D10 on D9 in contransfection. However, the translational advantage was not enhanced with a 1.3-fold increase (Fig 4B–4D) in which the D10 level was lower than when D10 was expressed alone (Fig 4A). The RNA levels were similar after 6 hours post transfection under different conditions (S6 Fig), suggesting the differences in luciferase activities were mostly due to translational difference of the RNAs. The results suggest additional mechanisms to coordinate D9 and D10 function during VACV infection. These mechanisms are under active investigation. This experimental setting likely underestimated the ability of D10 in promoting translation as the percentage of cells with both plasmid and RNA transfected was less than 100%. Exogenous expression of D9 is more similar to the overexpression of human Dcp2 that suppressed translation of both 12A-Fluc and Kozak-Rluc (S7 Fig). Thus, our data discovered a translational enhancement function of D10 in the absence of infection, with a stronger effect on poly(A)-leader-mediated translation than non-poly(A)-leader-mediated translation.

To further investigate whether the decapping activity is needed to enhance poly(A)-leader-mediated translation in virus-free condition, we generated D9 (E129Q, E130Q) and D10 (E144Q, E145Q) mutants with their catalytic site mutated to lose their decapping activities [25]. The result showed that D10mu could not stimulate poly(A) leader-mediated translation advantage of Fluc RNA (Fig 4A–4D). Together with the results that D9 and hDcp2 (S7 Fig) did not possess similar translation enhancement function, our results indicate that decapping activity of D10 is necessary but not sufficient to stimulate translation.

### Inactivation of VACV decapping enzymes impairs cap-independent translation enhancement during viral infection

**Fig 5:**
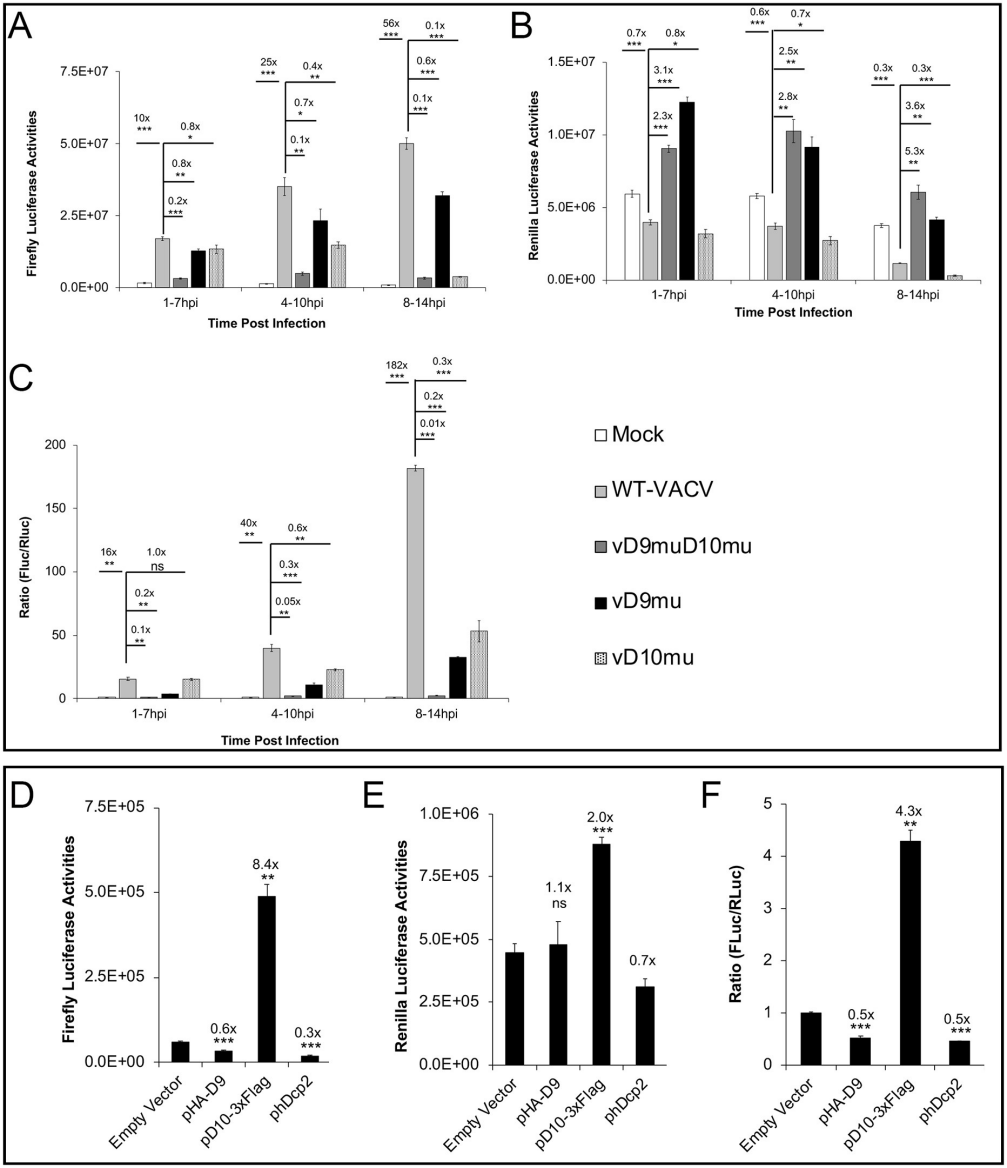
VACV decapping enzymes enhance cap-independent translation mediated by 5’-poly(A) leader. **(A-C)** Co-transfection of ApppG-capped 12A-Fluc and m^7^G-capped Kozak-Rluc in WT, vD9muD10mu, vD9mu, and vD0mu-infected A549DKO cells at indicated time post-infection. Luciferase activities were measured 6 h post-transfection. Fluc (**A**), Rluc (**B**), and Fluc/Rluc ratios with mock-infected samples normalized to 1 (**C**) are presented. **(D-F)** 293T cells were transfected with D9, D10, or human Dcp2 expression plasmid. 42 h post-transfection, in vitro synthesized, ApppG-capped 12A-Fluc, and m^7^G-capped Kozak-Rluc were co-transfected into the 293T cells. Luciferase activities were measured 6 h post RNA transfection. Fluc (**D**), Rluc (**E**), and Fluc/Rluc ratios with the empty vector normalized to 1 (**F**) are shown. Error bars represent the standard deviation of 3 replicates. Significance determined by students *t-test* where p>0.05 (ns), p≤0.05 (*), p≤0.01 (**), p≤0.001 (***). The numbers above significance represent fold changes.

Cap-independent translation enhancers (CITEs) are mRNA elements that promote cap-independent translation. CITEs may be located in the 5’- or 3’-UTRs [42]. Although not internal ribosome entry sites (IRESs), they facilitate recruiting translational machinery independent of cap-dependent translation initiation factors through mechanisms that are poorly understood. We and others have previously shown that a poly(A)-headed mRNA could be efficiently translated without the need of an m^7^G cap, as well as in VACV-infected cells with impaired cap-dependent translation [33, 43, 44]. These findings suggest that a poly(A)-leader is a CITE during infection. An ApppG-cap analog on an mRNA can protect mRNA from degradation but cannot initiate cap-dependent translation [45, 46]. Using an ApppG-capped 12A-Fluc mRNA co-transfected with an m^7^G-capped Kozak-Rluc, we found that inactivation of either D9 or D10 significantly decreased the ApppG-capped 12A-Rluc mRNA translation advantage conferred by VACV infection, and the inactivation of both decapping enzymes almost completely abolished the translational advantage during infection (Fig 5A–5C). We also observed an increasing translation enhancement of ApppG-capped 12A-Fluc with the progression of VACV replication (Fig 5A–5C). These findings indicate that D9 and D10 are necessary for VACV stimulation of cap-independent translation mediated by the 5’-poly(A) leader CITE.

### D10 expression promotes 5’-poly(A) leader-mediated cap-independent translation enhancement in uninfected cells

To further define the role of VACV decapping enzymes in stimulating cap-independent translation, we carried out these experiments similar to Fig 4B–4D except we used an ApppG-capped 12A-Fluc. We observed an 8.4-fold increase of 12A-Fluc activity in cells with D10 expression, while the m^7^G-capped Kozak-Rluc had a 2-fold increase (Fig 5D and 5E). The ratio of Fluc to Rluc indicated a 4.3-fold increase (Fig 5F). Neither D9 nor human Dcp2 enhanced 12A-Fluc mRNA translational advantage (Fig 5D–5F). Our results indicate that D10 promotes cap-independent translation of mRNA with a poly(A) leader.

### D9 and D10 do not associate with polysomes

**Fig 6:**
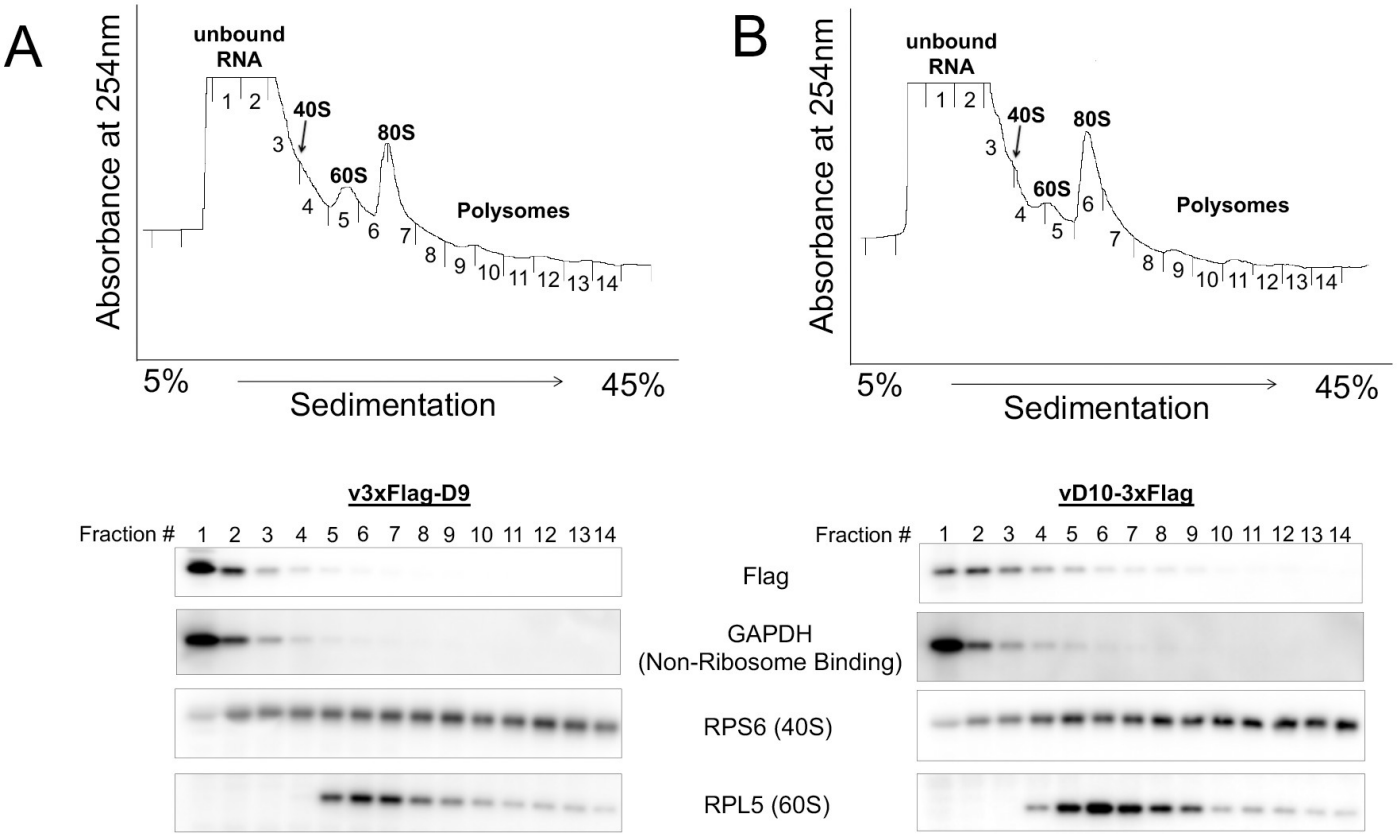
The majority of D9 and D10 do not associate with polysomes. **(AB)** A549DKO cells were infected with v3xFlag-D9 (**A**) or vD10-3xFlag (**B**) at an MOI of 5. Polysome profiling was carried out at 8 hpi. Proteins were detected in different fractions using indicated antibodies.

We examined if D9 or D10 associates with polysomes during VACV infection in A549DKO and HeLa cells. A polysome profiling followed by the detection of proteins in different fractions of the profiles indicated that the majority of the D9 and D10 co-sediment with the non-ribosome-bound fractions (Fig 6A and 6B and S8 Fig). Very little D9 or D10 is associated with the polysome or monosome fractions. The findings suggest that VACV decapping enzymes reprogram the cellular environment, but do not directly interact with the polysomes to affect mRNA translation during VACV infection.

## Discussion

Our findings reveal an unexpected function of VACV decapping enzymes that promote selective translation of viral mRNAs with a 5’-poly(A) leader. Messenger RNA decay and translation are two intertwined processes to regulate gene expression. Not surprisingly, activation of cellular decapping enzymes had been found to suppress mRNA translation through forming translation inhibitory P bodies or competing with translation initiation factors for binding to the 5’-cap structure [9, 10, 13, 14, 17–19, 47]. However, remarkably, here we found that D10 stimulates mRNA translation with a preference to RNA with a poly(A) leader both in the context of VACV infection and in uninfected cells. While D9 suppresses protein synthesis similar to eukaryotic Dcp2, it synergizes D10’s effect to promote viral post-replicative mRNA translation during VACV infection. Interestingly, although D9 and D10 have opposite effects on translation, both favor the translation of mRNA with a poly(A) leader over other non-poly(A) 5'-UTRs. During infection, a combination of D9 and D10 confers an optimal translation advantage to mRNA with a 5’-poly(A) leader. Therefore, although D9 and D10 induce viral and cellular mRNA degradation, they selectively promote viral post-replicative mRNA translation to advantage the synthesis of viral protein over cellular proteins during VACV infection.

**Fig 7:**
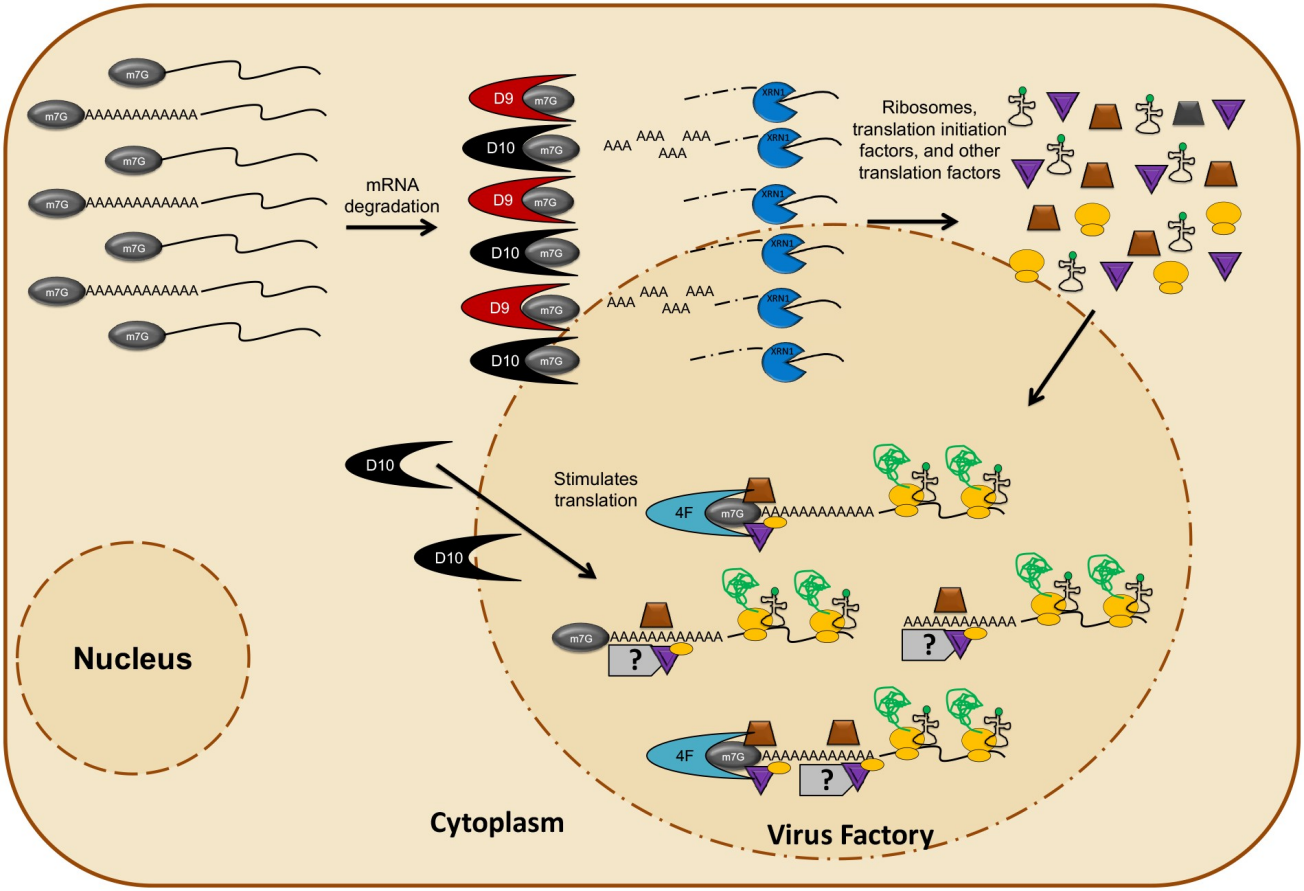
A proposed model of selective translational conferred to VACV post-replicative mRNA by viral decapping enzymes. D10 stimulates mRNA translation with a preference to 5’-poly(A)-headed mRNA during VACV infection. While not shown in the figure, D10 also stimulates translation in the absence of VACV infection. Promotion of 5’-poly(A) leader-mediated mRNA translation is amplified by D9 and D10-mediated RNA degradation during VACV infection. Cellular and viral mRNAs are decapped by D9 and D10, followed by Xrn1-mediated degradation. Degradation of mRNAs liberates ribosomes and other translation factors, which are recruited into the virus factory for translating viral post-replicative RNA by a coordination of the 5’-poly(A) leader-mediated cap-dependent and cap-independent translation. Actively translating mRNAs are mostly not accessible by D9 and D10.

Our data suggest molecular and cellular mechanisms for selective synthesis of viral proteins promoted by D9 and D10 during VACV infection (Fig 7). First, translation machinery is available for viral mRNA translation through D9- and D10-mediated degradation of cellular mRNAs in infected cells. The induction of mRNA degradation also avoids dsRNA-mediated translation arrest [22–26]. Second, D10 expression creates a cellular environment favoring active protein synthesis through its unexpected function to stimulate mRNA translation with a yet to be defined mechanism. Third, although D9 (suppression) and D10 (stimulation) have opposite effects on translation, together they synergize to achieve a translational advantage to viral mRNAs with 5’-poly(A) leaders. Finally, during VACV infection, there may be a spatial mechanism to prevent D9 and D10 from accessing actively translating mRNA (mainly VACV post-replicative mRNAs) and escape from decapping-mediated translation repression, as indicated by our polysome profiling in Fig 6. We are actively investigating these aspects.

The characterization of D10 as a translational activator is unexpected, as other decapping enzymes cause translation repression that involves the formation of processing bodies (PBs) or interfere with the translational process [17, 48–51]. One possibility why D10 does not repress translation is that its access to actively translating VACV mRNAs is limited during infection. VACV post-replicative mRNA transcription and translation occur in the “viral factory,” the site of viral replication that is characterized by intense extra-nuclear staining of viral DNA [52]. m^7^G-cap had been found on VACV post-replicative mRNAs’ 5’ ends [30]. The cap-recognition protein eIF4E and eIF4G scaffold protein of the cap-dependent translation initiation complex (eIF4F) are suggested to be recruited to viral factories to promote VACV post-replicative mRNA translation, at least in some cell types [22, 53–56]. It is possible that D10 and D9 are mostly not in the viral factories, which would give eIF4E a binding advantage to capped mRNAs over decapping enzymes. In addition, the cleaved 5’-m^7^G caps and their derived analogs from cellular and viral early mRNAs may sequester viral decapping enzymes, an idea supported by the feature of D10 compared to other decapping enzymes, in that it has a higher binding affinity to free cap and cap analogs [22, 57]. The limited accessibility to actively translating RNAs, plus the release of translational machinery due to the degradation of cellular mRNAs, can provide a mechanism for a general mRNA translational advantage.

Additional mechanisms are needed for viral decapping enzymes to confer selective translation of viral mRNA with a 5’-poly(A) leader as the limited accessibility cannot explain the observation that mRNA with a 5’-poly(A) leader has a higher translational advantage over mRNA without one. Enhanced recruitment of translation machinery by the 5'-poly(A) leader independent of a 5'-cap can provide a mechanism. In fact, some 5’-capped cellular mRNAs can use cap-independent translation initiation that contributes significantly to overall protein production in cell differentiation and under stress conditions [42]. VACV D9- and D10-induced accelerated mRNA degradation would liberate translational machinery to be more readily available for 5'-poly(A) leader-mediated cap-independent translation. Supporting this idea, an ApppG-capped poly(A)-headed mRNA translational advantage was reduced more than 20-fold in vD9muD10mu-infected cells than in WT VACV-infected cells (Fig 5C). Expression of D10 alone enhanced translation of ApppG-capped Fluc mRNA with a poly(A) leader (Fig 5D–5F). We hypothesize three possibilities by which the cap-independent translation enhancement by a poly(A) leader may contribute to the selective translation. First, a capped, poly(A)-headed mRNA can coordinate both cap-dependent and cap-independent translation mechanisms. Second, the 5’-poly(A) leader can efficiently carry out cap-independent translation mechanisms even in the presence of the m^7^G cap and cap-dependent initiation factors, while most other cellular 5’-UTRs cannot. Third, it is possible that there are non-capped, 5’-poly(A)-headed mRNAs in VACV-infected cells that can be actively translated. In fact, during the revision of this paper, a preprint manuscript was available, which suggests that some poly(A)-headed mRNAs in VACV-infected cells do not have a 5’-m^7^G cap [58].

Supporting the role of cap-independent translation in the selective translation of mRNA with a poly(A) leader, VACV infection stimulates a cap-independent translation cellular environment, as shown in our results (Fig 5A–5C). While both cap-dependent and cap-independent translation modes may operate during VACV infection, an increasing capability of cap-independent translation is observed in our previous work and confirmed in this study [33]. The cellular mRNAs are mostly degraded during VACV infection, which reduces the number of cellular competitors for viral mRNAs to access cap-dependent translation initiators. However, VACV also produces a large number of short RNAs with 5’-caps, which likely compete for cap-binding translation initiators [31, 37, 38, 59–61], and augment the importance of cap-independent translation. RACK1 is a specialized cellular 40S ribosomal protein dispensable for general cellular mRNA translation but critical for translation of VACV mRNA with a poly(A) leader [34]. Interestingly, RACK1 is important for cap-independent translation of RNAs of several RNA viruses containing IRESs [62, 63]. Although the 5'-poly(A) leader is not an IRES [33], its cap-independent translation enhancement may also utilize factors like RACK1 to promote the translation.

In summary, we identified VACV decapping enzymes as necessary factors to promote selective translation of viral mRNAs with a poly(A) leader. More intriguingly, we found that D10 is a translational activator. Decapping enzymes are a family of key gene expression regulators in eukaryotic cells with essential functions in many biological processes. They are also encoded in several other viruses. Our results not only provide a mechanism for selective translation of VACV mRNAs but also open an exceptional opportunity to dissect more broad and diverse roles of decapping enzymes in regulating gene expression through both mRNA decay and translation modulation.

## Materials and methods

### Cells and viruses

A549 DKO, kindly provided by Dr. Bernard Moss, 293T (ATCC-CRL-3216), and HeLa (ATCC CCL-2) cells were cultured in Dulbecco’s Modified Eagles Medium (Quality Biological). BS-C-1 (ATCC CCL-26) cells were cultured in Eagles Minimal Essential Medium (Quality Biological). All growth media was supplemented with 10% FBS (Peak Serum), 2 mM L-glutamine (Quality Biological), and 100 U/mL penicillin, 100 μg/mL streptomycin (Quality Biological). Cells were grown at 37°C and 5% CO2. VACV Western Reserve (WR) strain (ATCC VR-1354) and any derived recombinant viruses (except for vD9muD10mu) were grown in HeLa cells and purified on 36% sucrose gradient and titrated by plaque assay as described elsewhere [64]. Titration of vD9muD10mu was grown and titrated in A549DKO cells as described elsewhere using anti-VACV antibody and HRP conjugated secondary antibody [25, 64].

### Recombinant VACV generation

vD9mu, vD10mu, and vD9muD10mu were kindly provided by Dr. Bernard Moss. Generation of vD10mu was described by Liu et al. [24], while vD9mu and vD9muD10mu were described elsewhere [25]. To generate v3xFLAG-D9 or vHA-D9, eGFP under VACV late promoter P11 was first inserted in place of the D9R ORF from VACV Western Reserve Strain (ATCC VR-1354), and then D9R with 3xFLAG or HA peptide coding sequence attached to the 5’-end of D9 CDS was inserted in place of eGFP. vD10-3xFLAG was generated first by attaching the 3xFLAG sequence to the 3’-end of D10R CDS, and an eGFP under the P11 promoter was used for selection of the recombinant virus; the eGFP sequence was then removed. Those modified sections of the viral genome were amplified by PCR and sequenced for confirmation.

### Antibodies and chemicals

Anti-FLAG mouse monoclonal antibody was purchased from Stratagene (Cat # 200472). Anti-HA mouse monoclonal antibody was purchased from Thermo Fisher Scientific (Cat #26183). Anti-GAPDH, HRP-conjugated antibody was purchased from Santa Cruz Biotechnologies (Cat #sc-365062 HRP). Goat anti-mouse and anti-rabbit HRP conjugated secondary antibodies were purchased from Azure Biosystems (Cat #AC2115) or Cell Signaling Technologies (Cat #7074S), respectively. Antibodies raised against VACV proteins A17 and A10 were gifts from Dr. Bernard Moss [65–67]. Antibodies raised against VACV protein E3 were a gift from Dr. Yan Xiang [68]. Cycloheximide(Cat#01810) was purchased from Sigma. Phenol:Chloroform:Isoamyl Alcohol (Cat#15593031) was purchased from Invitrogen. qPCR mix (Cat#QP005) was purchased from GeneCopoeia. SuperScript III Reverse Transcriptase (Cat#18-080-044), Trizol reagent (Cat#15-596-018), RNase inhibitor (Cat#AM2696), DTT (Cat#BP172) and D-Sucrose (Cat#BP220-212) were purchased from Fisher Scientific.

### Polysome profiling

The method was adopted elsewhere with modifications [69]. Cells in T175 cell culture flasks were infected with WT-VACV or vD9muD10mu at an MOI of 5 with three biological repeats. At 8hpi, cell culture medium was replaced with fresh medium (containing 100 μM CHX) and incubated for 20 min at 37°C. The medium was then replaced with cold PBS (containing 100 μM CHX), and cells were scraped off from the cell culture flasks. Cell pellets were collected by centrifuging at 500xg for 5min at 4°C. Cell pellets were resuspended in 300μL of cold hypotonic lysis buffer (containing 5 mM pH7.5 Tris-cl, 2.5 mM MgCl_2_, 1.5 mM KCl, 100 μg/mL CHX, 1 mM DTT, 1% Triton X-100, 40 U/mL RNase inhibitor, 1x protease inhibitor cocktail) and rotated at 4°C for 30 min before being centrifuged at 12,000xg for 10 min at 4°C. The supernatants were loaded on top of sucrose gradients (from 5% to 45%, made with buffer containing 20 mM pH7.5 Tris-Cl, 50 mM KCl, 10 mM MgCl_2_, 1 mM DTT, 100 mg/mL CHX, 20 U/mL RNase inhibitor) and centrifuged at 38,000xg for 90 min at 4°C. The layered polysome components were separated and collected with a gradient fractionator (BR-188, Brandel). One volume of Trizol was added to RNA-containing sucrose right after they were collected. The fractions containing non-ribosome-binding RNAs and ribosome-binding RNAs were pooled together for RNA extraction, respectively.

### RNA extraction

Trizol-containing samples collected from polysome profiling step were mixed with 1/5 volume of Chloroform. The mixtures were vortexed and set at room temperature for 15 min. Then the mixtures were centrifuged at 15,000xg for 10 min at 4°C. The RNA-containing top clear potion was transferred to a new tube and mixed with 1 volume of isopropanol and 1/10 volume of Sodium Acetate Solution (pH5.2). The mixtures were incubated at -20°C for 1 h before being centrifuged at 15,000xg for 10 min at 4°C to precipitate the RNA. To improve RNA quality, RNA pellets were dissolved in RNase-free water followed by addition of 3/5 volume of Phenol:Chloroform:Isoamyl Alcohol (25:24:1, v/v/v). The mixture was vortexed and set at room temperature for 15 min. The mixture was centrifuged at 15,000xg for 10 min at 4°C. The top clear layer was transferred to a new tube, and the isopropanol precipitation of RNA, as described above, was repeated to obtain RNA pellets. The RNA pellets were washed with 75% ethanol and centrifuged at 15,000xg for 10 min at 4°C. After removing 75% ethanol, the RNA pellets were semi-dried, dissolved using RNase-free water, and measured for concentration and quality using Bioanalyzer.

### RNA-Seq and analysis

Samples of extracted RNA were sequenced in the Integrated Genomics Facility at Kansas State University for a paired-end sequencing. About 1 million reads were obtained for each sample. The sequencing data were analyzed using, as described previously [70, 71]. RPKM of cellular genes and VACV genes was calculated for each sample. Relative translation efficiency of each mRNA was calculated by comparing RPKMs of ribosome-binding RNA to non-ribosome-binding RNA. RNA-Seq data is deposited to NCBI SRA under the BioProject accession number PRJNA656284.

### Quantitative RT-PCR (qRT-PCR) and Western blotting analysis

For qRT-PCR test, infected cells were collected in Trizol to extract total RNA. RNA was reverse transcribed to cDNA with SuperScript^™^ III Reverse Transcriptase (Thermo Fisher Scientific, Cat#18080093) using random hexamers. qPCR was carried out with specific primers for individual genes. 18s RNA was used as the normalization control. For Western Blotting analysis, cells were collected in 1X RIPA cell lysis buffer and rotated at 4°C for 30 min. After being centrifuged at 12,000xg for 10 min at 4°C. The supernatants were mixed with SDS loading buffer and boiled for 5 min. The samples were separated by SDS-PAGE and analyzed with specific antibodies.

### Codon optimization, plasmid construction, and transfection

Vectors expressing decapping enzymes (pcDNA3.1-HA-D9, pcDNA3.1-D10-3xFlag, pcdNA3.1-D9mu-3xFlag, pcDNA3.1-D10mu-3xFlag) were codon-optimized for expression in human cells. These plasmids, in addition to the vector containing the gene for human Dcp2 (hDcp2), not requiring optimization, were manufactured by GenScript. Briefly, genes were cloned into pcDNA3.1 (-) (Thermo Fisher Scientific) downstream from the CMV promoter using 5’ and 3’ restriction sites of KpnI and HindIII, respectively. Plasmids were grown in NEB 5-α competent *E*. *Coli* (New England Biolabs), purified using E.Z.N.A. Plasmid DNA Mini Kit (Omega Bio-Tek), quantified using Nanodrop (Thermo Fisher Scientific), and transfected into cells using Lipofectamine 3000 (Thermo Fisher Scientific) in accordance with the manufacturer’s instructions.

### *In vitro* RNA synthesis and luciferase assay

Synthesis of RNA *in vitro* and Luciferase assays were performed as previously described [33, 40]. Briefly, primers were designed to amplify DNA fragments that included the T7 promoter, desired 5’UTR, reporter gene (Firefly luciferase or *Renilla* luciferase), and a poly(A) tail using Q5 High Fidelity DNA polymerase (New England Biolabs, Cat#M0492L). DNA was purified using E.Z.N.A. Cycle Pure Kit (Omega Bio-Tek, Cat# D6492-01) and the concentration determined by Nanodrop. RNA was synthesized from newly amplified DNA template using HiScribe T7 Quick High Yield RNA Synthesis Kit (New England Biolabs, Cat#E2050) and co-transcriptionally capped with m^7^G anti-reverse cap analog (ARCA, Cat#1411) or ApppG Cap Analog (New England Biolabs, Cat#1406) according to the manufacturer’s protocol. Reporter RNA was purified using a Purelink RNA Mini Kit (Thermo Fisher Scientific, Cat#12183025) and measured using Nanodrop. At the desired experimental time, reporter RNA was transfected into cells in Opti-MEM (Thermo Fisher Scientific, Cat#31985062) using Lipofectamine 2000 (Thermo Fisher Scientific, Cat# L11668019) according to the manufacturer’s instructions. Six hours post-transfection, cell lysates were collected, and luciferase activities measured using a Dual Luciferase Reporter Assay System (Promega, Cat#E1960) and GloMax Navigator Microplate Luminometer with dual injectors (Promega) as per manufacturer protocol.

### Preparation of polysome profiling fractions for Western blotting analysis

Lysate from A549 DKO or HeLa cells infected with v3xFlag-D9 or vD10-3xFlag at an MOI of 5 was prepared for polysomes profiling as described above. Protein was precipitated from fractions collected during polysome profiling by addition of 2X volume 100% molecular biology grade ethanol to each fraction and storing at -80°C overnight. Precipitated protein was pelleted at 25,000xg at 4°C for 1 h, then washed with 70% ethanol and pelleted at 25,000xg for 5min at 4°C. Protein pellet was air-dried and resuspended and heated in 1X SDS sample buffer.

### Statistical analysis

Differences in luciferase activities as well as in polysome profiling and qRT-PCR were determined by the Students *t-test* where ns indicates not significant, * indicates p ≤ 0.05, ** indicates p ≤ 0.01, *** indicates p ≤ 0.001, and **** indicates p ≤ 0.0001. All error bars shown represent the standard deviation of three replicates.

## Supporting information

S1 Fig PKR and RNase L are not detected in A549DKO cells. Western blotting analysis was carried out to detect PKR and RNase L proteins in A549 and A549DKO cells using indicated antibodies.
(TIF)

S2 Fig Boxplot comparison of the lengths of VACV and cellular gene CDSs. The median (50%), Q1 (25%), and Q3 (75%) of human genes are 1083, 459, and 3422 nts. The median (50%), Q1 (25%), and Q3 (75%) of VACV genes are 642, 387, and 999 nts. **** indicates p≤0.0001.
(TIF)

S3 Fig Correlation co-efficiencies of biological replicates of ribosome-binding (A), and non-polysome binding (B) RNA-Seq. Only those genes with an RPKM>0.5 were used in the analyses.
(TIF)

S4 Fig Transfected RNA levels are similar in mock-, WT-VACV-, and vD9muD10mu-infected cells at time of luciferase assays. Quantitative RT-PCR were carried out to measure the transfected Fluc and Rluc RNA levels, respectively, after 6 h post-transfection of RNA into A549DKO cells with indicated virus (or mock) infection at **(A)** 1, **(B)** 4, or **(C)** 8 hpi. Results were an average of three biological replicates. The RNA level in mock-infected cells was normalized as 100%. Significance was determined by students *t-test*. No significant difference was detected between RNAs under any two conditions.
(TIF)

S5 Fig Translation of RNA with or without 12A’s as 5’-UTR. **(A-C)** Co-transfection of m^7^G-capped Fluc RNA with or without a poly(A) leader together with Kozak-Rluc RNA in mock, WT-VACV-, and D9muD10mu-infected A549DKO cells at 8 hpi, respectively. Luciferase activities were measured 6 h post-transfection. Fluc (**A**), Rluc (**B**), and Fluc/Rluc ratios with mock-infected samples normalized to 1 (**C**) are shown. Error bars represent the standard deviation of 3 biological replicates. Significance determined by students *t-test* where p>0.05 (ns), p≤ 0.05 (*), p≤0.01 (**), and p≤0.001 (***). Numbers above significance represent fold change between compared samples.
(TIF)

S6 Fig Transfected RNA levels are not significantly different in cells transfected with indicated plasmids at time of luciferase assays. Quantitative RT-PCR were carried out to measure transfected Fluc and Rluc RNA levels, respectively, after 6 h post-transfection of RNA into 293T cells with indicated plasmid transfected into the cells prior to RNA transfection. Results were an average of three biological repeats. The RNA level in mock-infected cells was normalized as 100%. Significance was determined by students *t-test*. No significant difference was detected between RNAs under any two conditions.
(TIF)

S7 Fig Comparison of the effects of D9, D10, and hDcp2 expression in uninfected cells on mRNA translation. **(A-C)** 293T cells were transfected with indicated plasmids. 42 h post-transfection, *in vitro* synthesized, m^7^G-capped 12A-Fluc, and Kozak-Rluc were co-transfected into the 293T cells. Luciferase activities were measured 6 h post RNA transfection. Fluc (**A**), Rluc (**B**), and Fluc/Rluc ratios with the empty vector normalized to 1 (**C**) are presented. Error bars represent the standard deviation of 3 replicates. Significance determined by students *t-test* where p>0.05 (ns), p≤0.05 (*), p≤0.01 (**), p≤0.001 (***). The numbers above significance represent fold changes. Significance and fold change were compared to the empty vector.
(TIF)

S8 Fig The majority of D9 and D10 do not associate with polysomes. **(AB)** HeLa cells were infected with v3xFlag-D9 (**A**) or vD10-3xFlag (**B**) at an MOI of 5. Polysome profiling was carried out at 8 hpi. Proteins were detected in different fractions using indicated antibodies.
(TIF)
