# Supplementary figures and images for: Poxvirus-encoded decapping enzymes promote selective translation of viral mRNAs
Source: PLoS Pathog. 2020 Oct 8;16(10):e1008926. doi: 10.1371/journal.ppat.1008926 (PMC7575113; doi:10.1371/journal.ppat.1008926)

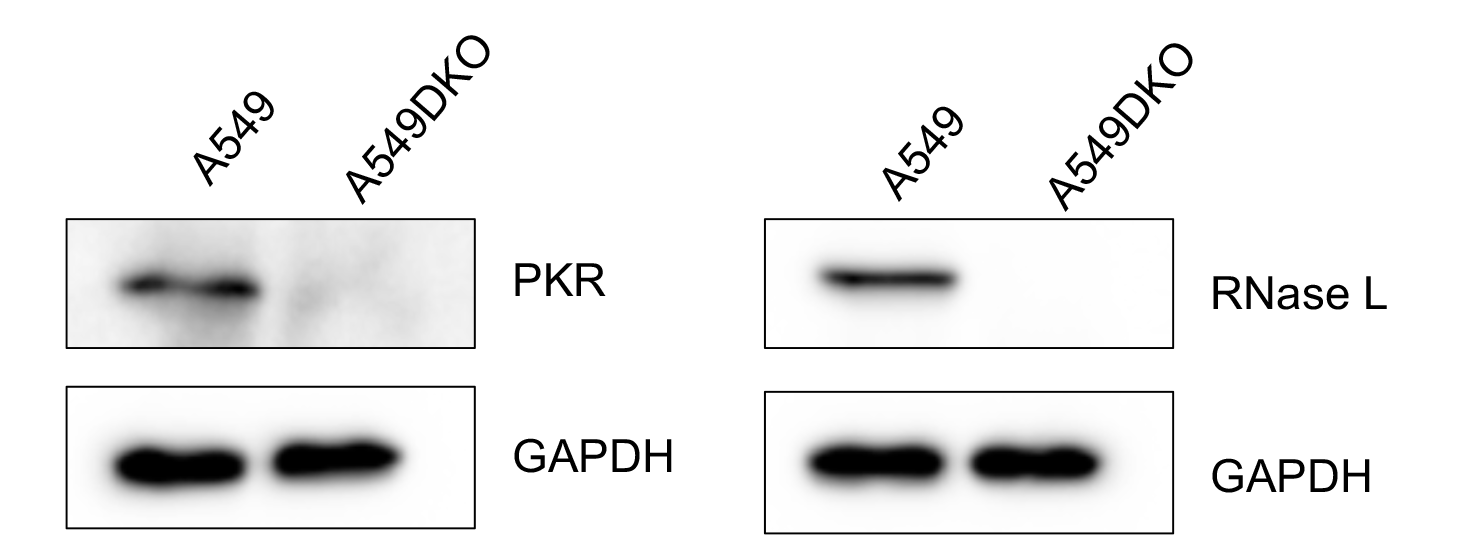

Supplement: S1 Fig — Western blotting analysis was carried out to detect PKR and RNase L proteins in A549 and A549DKO cells using indicated antibodies. (TIF) [file ppat.1008926.s001.tif]

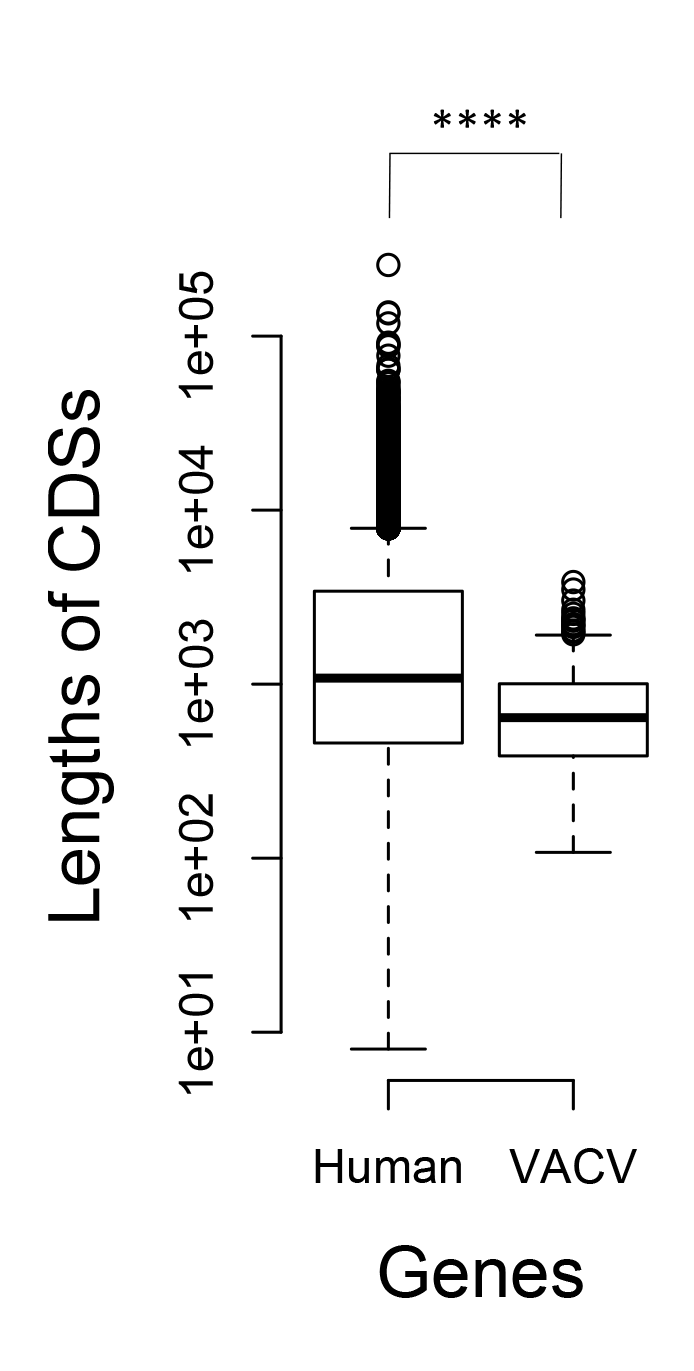

Supplement: S2 Fig — The median (50%), Q1 (25%), and Q3 (75%) of human genes are 1083, 459, and 3422 nts. The median (50%), Q1 (25%), and Q3 (75%) of VACV genes are 642, 387, and 999 nts. **** indicates p≤0.0001. (TIF) [file ppat.1008926.s002.tif]

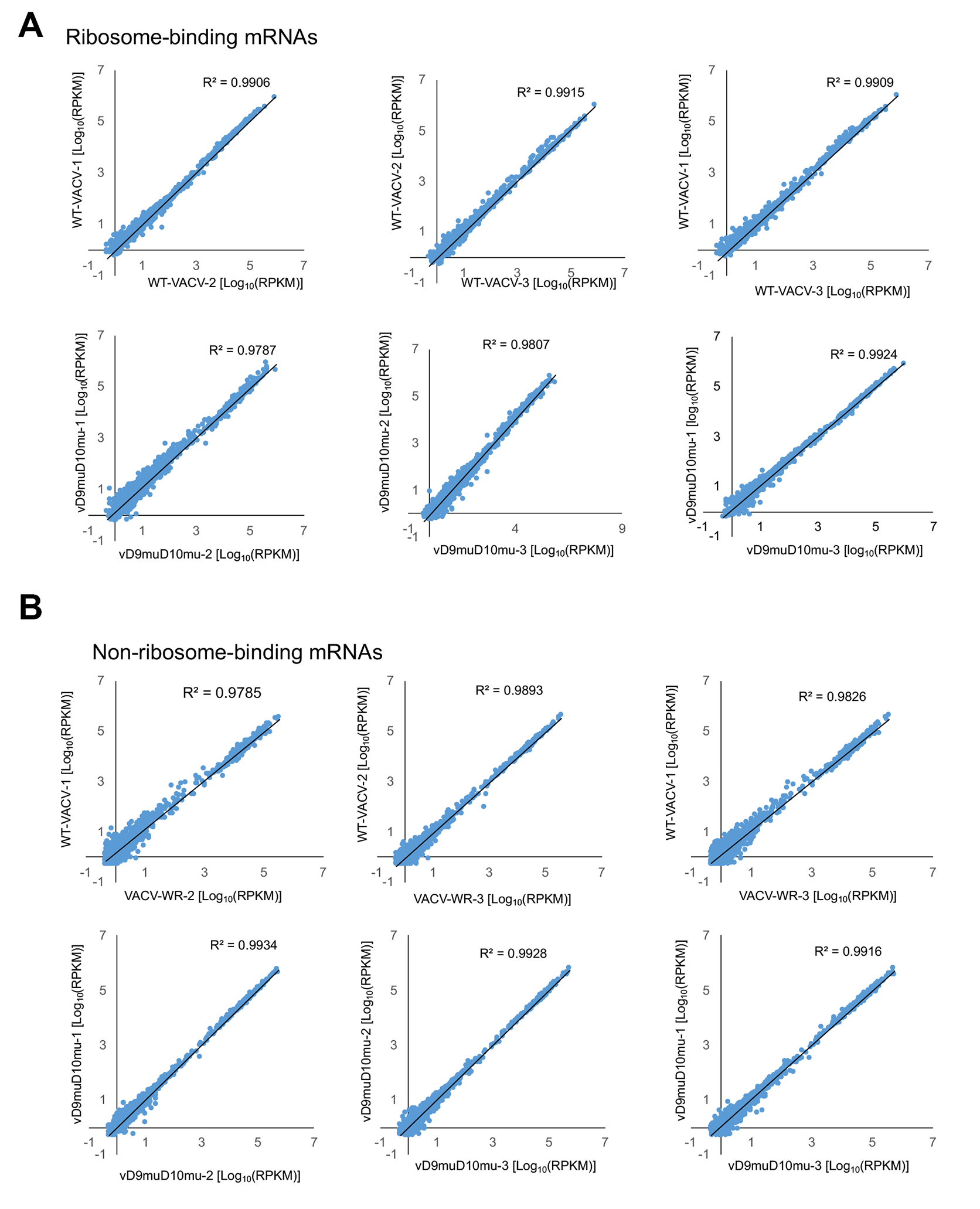

Supplement: S3 Fig — Only those genes with an RPKM>0.5 were used in the analyses. (TIF) [file ppat.1008926.s003.tif]

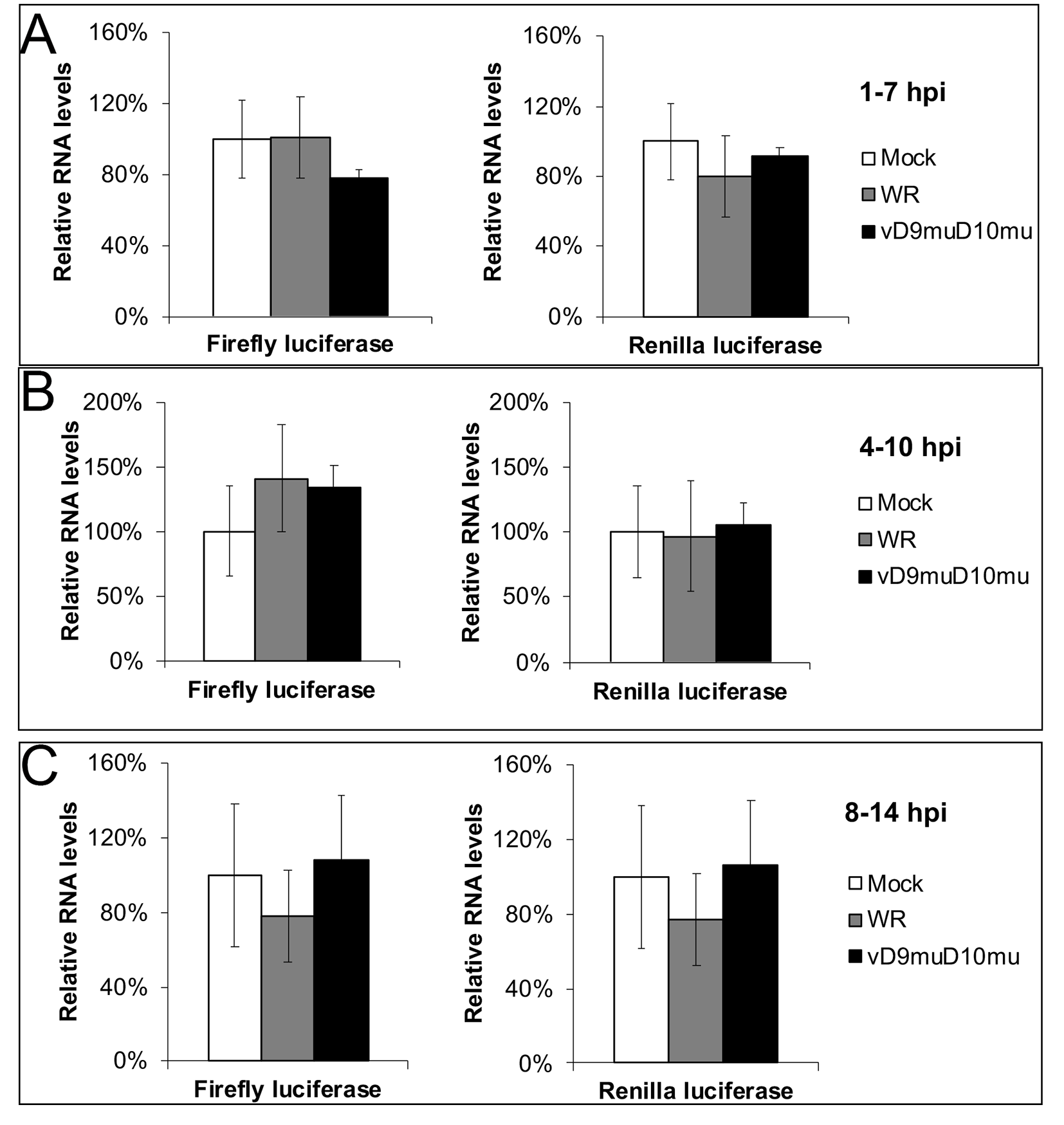

Supplement: S4 Fig — Quantitative RT-PCR were carried out to measure the transfected Fluc and Rluc RNA levels, respectively, after 6 h post-transfection of RNA into A549DKO cells with indicated virus (or mock) infection at (A) 1, (B) 4, or (C) 8 hpi. Results were an average of three biological replicates. The RNA level in mock-infected cells was normalized as 100%. Significance was determined by students t-test. No significant difference was detected between RNAs under any two conditions. (TIF) [file ppat.1008926.s004.tif]

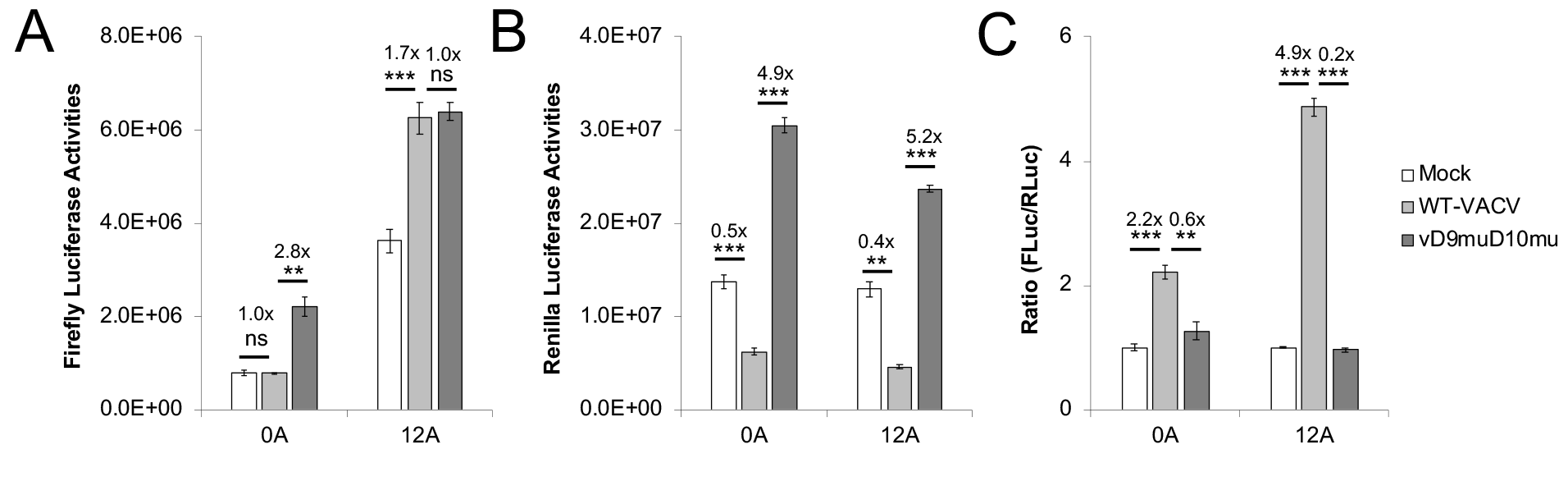

Supplement: S5 Fig — (A-C) Co-transfection of m7G-capped Fluc RNA with or without a poly(A) leader together with Kozak-Rluc RNA in mock, WT-VACV-, and D9muD10mu-infected A549DKO cells at 8 hpi, respectively. Luciferase activities were measured 6 h post-transfection. Fluc (A), Rluc (B), and Fluc/Rluc ratios with mock-infected samples normalized to 1 (C) are shown. Error bars represent the standard deviation of 3 biological replicates. Significance determined by students t-test where p>0.05 (ns), p≤ 0.05 (*), p≤0.01 (**), and p≤0.001 (***). Numbers above significance represent fold change between compared samples. (TIF) [file ppat.1008926.s005.tif]

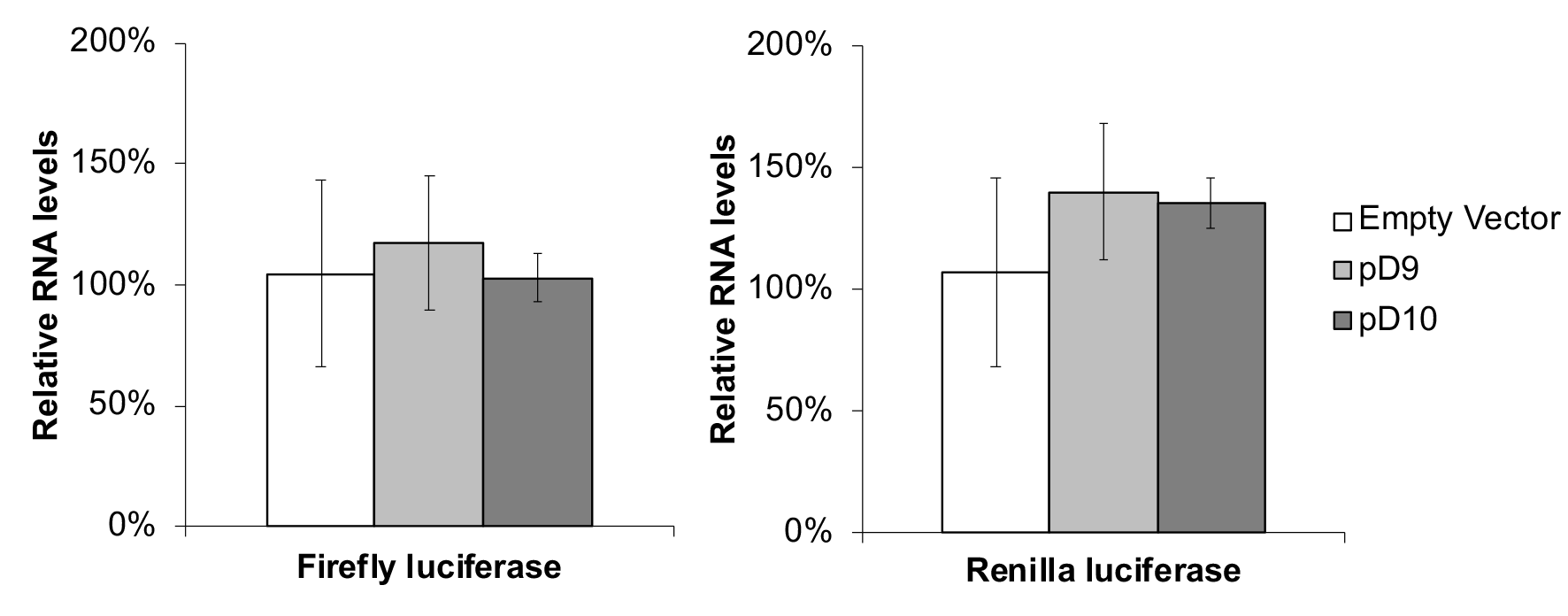

Supplement: S6 Fig — Quantitative RT-PCR were carried out to measure transfected Fluc and Rluc RNA levels, respectively, after 6 h post-transfection of RNA into 293T cells with indicated plasmid transfected into the cells prior to RNA transfection. Results were an average of three biological repeats. The RNA level in mock-infected cells was normalized as 100%. Significance was determined by students t-test. No significant difference was detected between RNAs under any two conditions. (TIF) [file ppat.1008926.s006.tif]

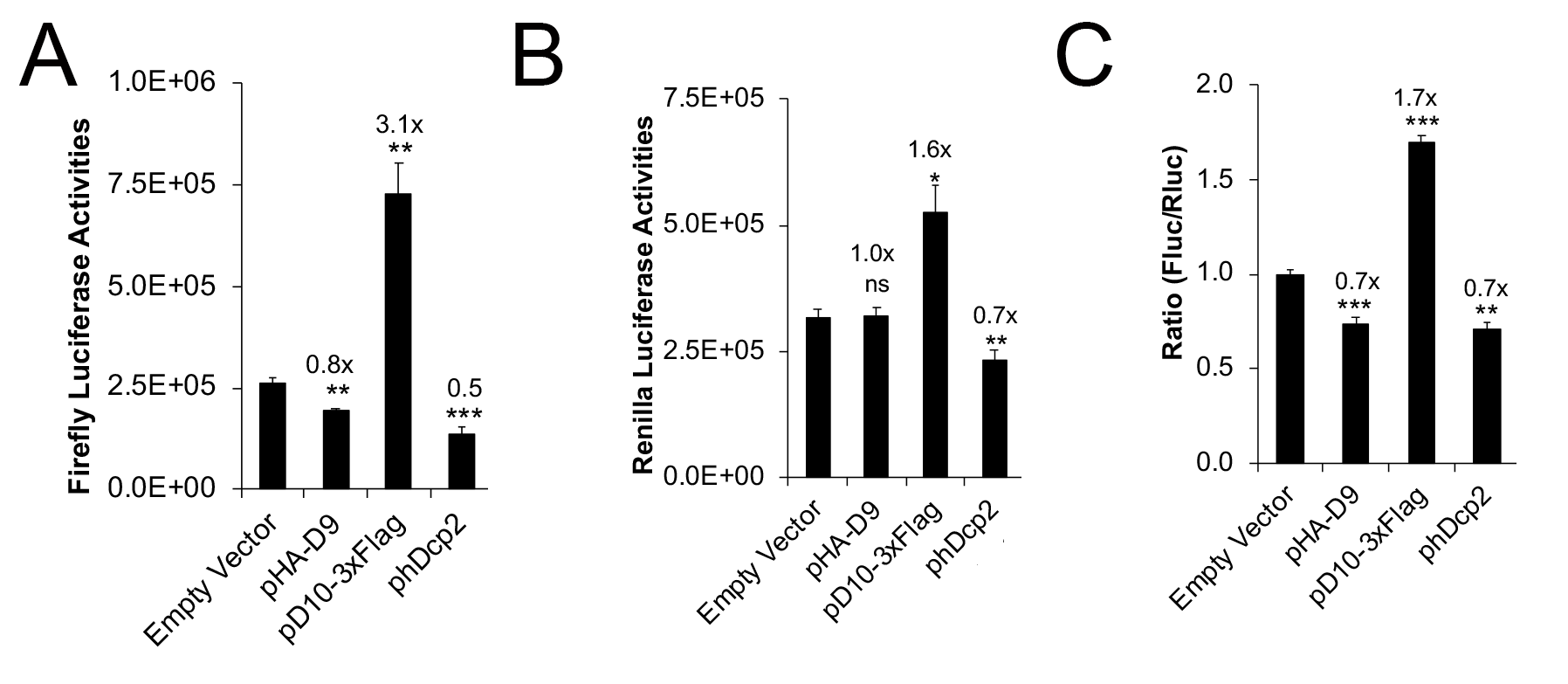

Supplement: S7 Fig — (A-C) 293T cells were transfected with indicated plasmids. 42 h post-transfection, in vitro synthesized, m7G-capped 12A-Fluc, and Kozak-Rluc were co-transfected into the 293T cells. Luciferase activities were measured 6 h post RNA transfection. Fluc (A), Rluc (B), and Fluc/Rluc ratios with the empty vector normalized to 1 (C) are presented. Error bars represent the standard deviation of 3 replicates. Significance determined by students t-test where p>0.05 (ns), p≤0.05 (*), p≤0.01 (**), p≤0.001 (***). The numbers above significance represent fold changes. Significance and fold change were compared to the empty vector. (TIF) [file ppat.1008926.s007.tif]

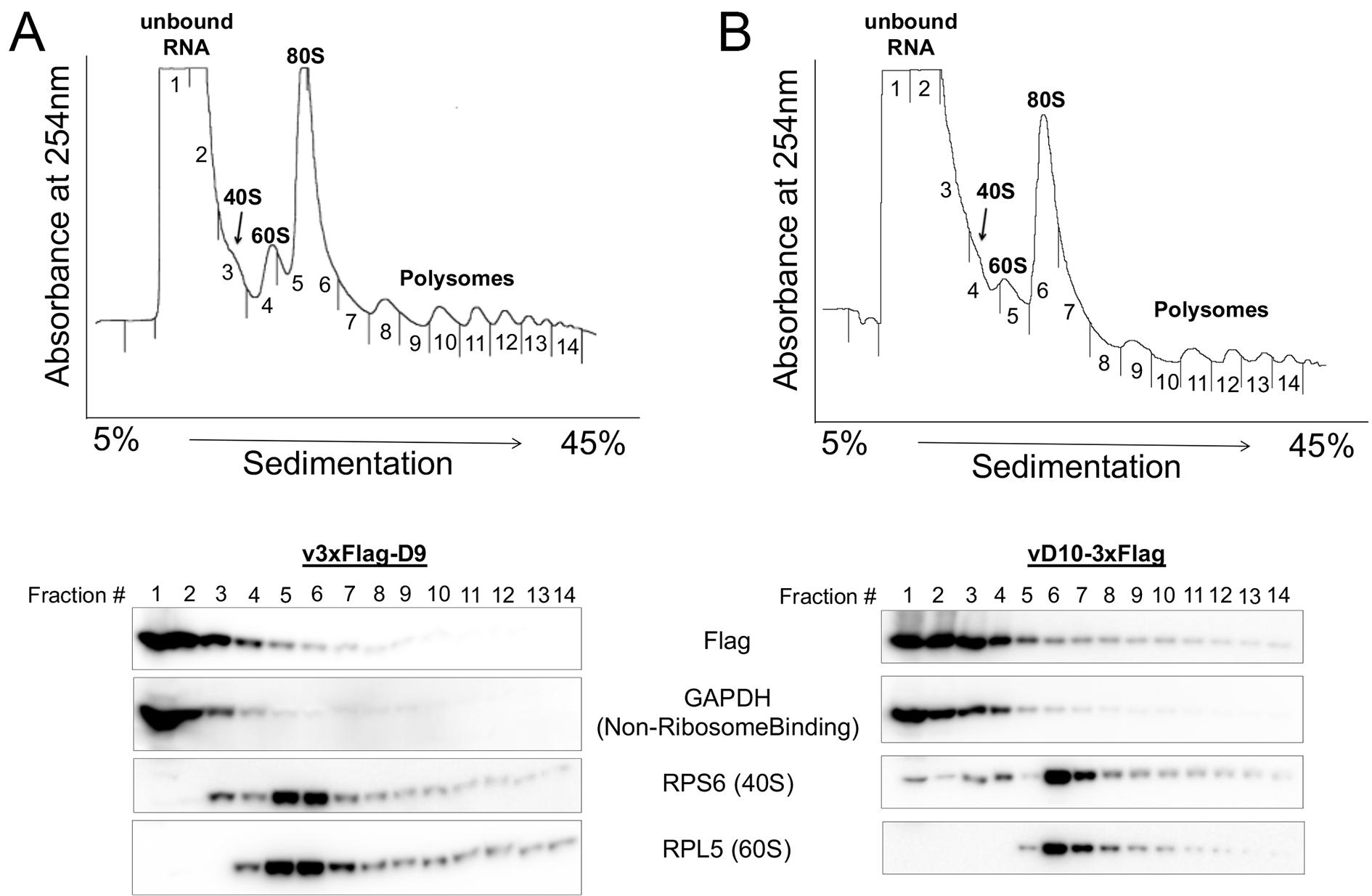

Supplement: S8 Fig — (AB) HeLa cells were infected with v3xFlag-D9 (A) or vD10-3xFlag (B) at an MOI of 5. Polysome profiling was carried out at 8 hpi. Proteins were detected in different fractions using indicated antibodies. (TIF) [file ppat.1008926.s008.tif]
